# A Multi-Functional Tubulovesicular Network as the Ancestral Eukaryotic Endomembrane System

**DOI:** 10.3390/biology4020264

**Published:** 2015-03-24

**Authors:** Juan Carlos González-Sánchez, Ricardo Costa, Damien P. Devos

**Affiliations:** Centro Andaluz de Biologia del Desarollo (CABD), Universidad Pablo de Olavide, Carretera de Utrera km 1, Seville 41013, Spain; E-Mails: jcgonsan@upo.es (J.C.G.-S.); rjdoscos@upo.es (R.C.)

**Keywords:** Eukaryogenesis, membrane system, evolution, hypothesis

## Abstract

The origin of the eukaryotic endomembrane system is still the subject of much speculation. We argue that the combination of two recent hypotheses addressing the eukaryotic endomembrane’s early evolution supports the possibility that the ancestral membranes were organised as a multi-functional tubulovesicular network. One of the potential selective advantages provided by this organisation was the capacity to perform endocytosis. This possibility is illustrated by membrane organisations observed in current organisms in the three domains of life. Based on this, we propose a coherent model of autogenous eukaryotic endomembrane system evolution in which mitochondria are involved at a late stage.

## 1. Introduction

Eukaryogenesis, the origin of the eukaryotic cell, is considered to be one of the major transitions in evolution. The cellular and molecular details of how this happened are still unknown. Molecular paleontology has taught us a great deal about the origin and early evolution of the eukaryotes [1]. It is now well-supported that the first eukaryotic cell (also known as the Last Eukaryotic Common Ancestor, LECA) already possessed most of the sophistication of modern eukaryotes, *i.e.*, linear chromosomes with telomeres, implying the presence of the proteins and structures to define those chromosomes; introns; the molecular systems for replication and transcription of the DNA and for RNA transcripts processing; cytoskeletal elements and associated motor systems; division by mitosis; phagocytosis; signalling systems, including kinase-phosphatase-based and ubiquitin-based machineries; and a complex endomembrane system (ES) resolved in spatially separated and functionally differentiated compartments, in addition to the mitochondria [1,2]. The order of appearance of these features, or if they have appeared concurrently, is however unknown. It is clear that it is the acquisition of the mitochondria as well as of a complex and functionally differentiated ES, including the nucleus, that defined the birth of the first eukaryotic cell.

## 2. Literature Review

Amongst these eukaryotic defining features, the impressively developed ES stands out. Because the ES plays such a central role in eukaryotic biology by allowing the functional differentiation of the cellular volume, understanding its origin is key to deciphering the origin of the eukaryotes. The eukaryotic ES is classically presented as composed of multiple spatially and functionally separated organelles, including the nucleus, the endoplasmic reticulum (ER), the Golgi apparatus, peroxisomes, and lysosomes. Eukaryotic organelles are of two types: those with a bacterial endosymbiotic origin, such as mitochondria, and chloroplasts in photosynthetic eukaryotes, and those derived from an autogenous (non-endosymbiotic) process, such as the Golgi apparatus or the ER. Much is known about the origin of endosymbiotic organelles (e.g., [3,4]). In contrast, our knowledge of autogenous organelles origin is more rudimentary. The fact that much of the molecular machinery involved in conferring specificity and function to membrane trafficking is evolutionarily ancient is increasingly supported [1,5]. In addition, phylogenetic reconstruction resolved around half of the Rab paralogs into two large clades corresponding to broadly endocytic and exocytic functions [6]. This implies that one of the earliest functional differentiations in the trafficking system was into ‘in’ and ‘out’ pathways, and this may have predated the emergence of many of the individual organelles.

Previously, most models of non-endosymbiotic organelles development were embedded in broader hypotheses and did not address ES origin thoroughly. Recently, two theories have enriched our understanding of eukaryotic ES evolution: the organelle paralogy (OPH) and protocoatomer hypotheses (PCH). The PCH states that the acquisition of the membrane coat (MC) proteins was key to the development of the eukaryotic ES by allowing early organisms to manipulate their membranes [7]. The OPH proposes that novel autogenous organelles arose as the result of gene duplication and neofunctionalization of pre-existing trafficking machinery [8].

We argue that the unification of these two related theories supports an undifferentiated tubulovesicular network (TVN) as a possible ancestral state of the eukaryotic ES. We comment on current TVN to speculate about the organization and function that such an ancestral ES might have taken. Based on this, we propose a scenario for autogenous evolution of the eukaryotic ES.

### 2.1 The Combination of the Organelle Paralogy and Protocoatomer Hypotheses Explains Autogenous Organelle Evolution

Functionally differentiated eukaryotic organelles are tightly linked to each other, either by direct membrane contacts [9] or by a vesicle trafficking system [10]. These compartments maintain a different internal chemical composition necessary for their functional diversification via a dynamic transport system that is mostly vesicle-mediated. Well-characterized sets of protein machineries are involved in the correct functioning and maintenance of each of the organelles, including GTPases, adaptors, and tethering complexes [10]. Organelle identity is the product of the combinatorial interaction of the trafficking proteins found at a particular organelle. Many of these proteins belong to paralogous families, *i.e.*, they are the result of gene duplication and divergence. Not surprisingly, proteins belonging to the same family have similar roles in different organelles. Examples include the adaptors protein complexes of membrane coated vesicles [11] and the Rab family of GTPases that regulate similar steps of membrane traffic between different organelles [6,12].

#### 2.1.1. The Organelle Paralogy Hypothesis

Phylogenetic analyses of the paralogous gene families in contact with the eukaryotic ES, such as Rabs, syntaxins, adaptins, and Arf-GAPs, have shown that most of the gene duplications predate the first eukaryotic cell. However, some lineage specific expansions of endocytic proteins were observed indicating a later origin, *i.e.*, they appeared after the primary eukaryote radiation into the major supergroups [6,13,14,15,16,17]. Examples include Rab5 independent paralogous expansion in humans and trypanosomes, or SNARE proteins Vam3/Pep12 *vs.* Syntaxins 7 and 13, as well as β1 and β2 subunits of the adaptin complex [8,18]. These examples demonstrate parallel but independent evolution of the endocytic machinery. Thus, organelles in those organisms are paralogues, as resulting from lineage specific expansions.

**Figure 1:**
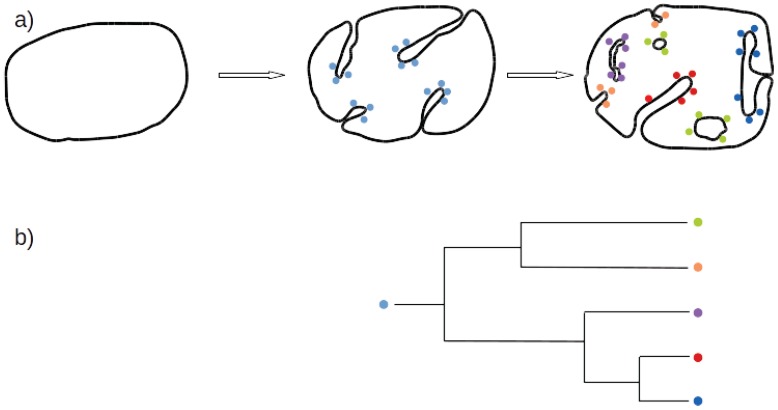
Early evolution of the endomembrane system, paralogous expansion and evolution of membrane manipulation complexes. (**a**) Early evolution of the endomembrane system can be divided into three steps: (1) a prokaryote without additional features to its external membrane other than surrounding it; (2) acquisition of multi-protein complexes for membrane manipulation (e.g., MC proteins) allowed this organism to invaginate and manipulate its membrane; and 3) duplication and evolutionary divergence led to spatial separation and functional differentiation of the compartments formed. For clarity, only the MC proteins are represented (coloured spheres). Proteins of the same family have the same colour; (**b**) Illustrative-only phylogenetic tree of MC proteins. After the initial birth of this protein (blue sphere), duplication and divergence lead to different protein families (coloured spheres).

The OPH proposes that the diversification of the various current eukaryotic ESs was caused by iterative gene duplications, followed by sequence divergence and neofunctionalization in multiple interacting proteins determining organelle identity and pathway specificity [8,19]. Increases in the complexity of specificity-encoding protein families are mirrored by increases in the complexity of the membrane-trafficking system (Figure 1). Computer simulations using a biophysical model of eukaryotic endomembrane evolution support the OPH [20].

This hypothesis therefore implies earlier states of development with an increasingly reduced number of genes and fewer spatially separated compartments, potentially functionally less diversified. Duplication and divergence of those genes led to the multiplication of membrane structures and the separation of function in the newly formed compartments.

#### 2.1.2. The Protocoatomer Hypothesis

The PCH, in complete agreement with the OPH, recapitulates the evolutionary history of the membrane coat (MC) proteins [7] (Figure 1). MC proteins are central to the eukaryotic trafficking system because they form the scaffold of the multi-protein complexes surrounding the membrane vesicles which ensure trafficking between compartments. As originally defined by the PCH, MC proteins included only some coated vesicles and nuclear pore complex components, like the clathrin heavy chain, COPI α and β' subunits, and COPII Sec31 or Nup85 and Nup133 (using the yeast protein names) [7]. Recent analyses have expanded this hypothesis by revealing the presence of MC proteins in transport of materials along a cilia’s or flagella’s microtubules in TSET, an ancient component of the eukaryotic membrane-trafficking system, and in the SEA and HOPS/CORVET complexes, whose functionalities in signalling and trafficking are still unclear [21,22,23,24].

Shared structural features suggest a related origin for all MC proteins and by extension for their multi-protein complexes and the compartments that they sustain [7]. Subsequent structural studies strongly support the similarities observed between those proteins, further reducing the chances that these complexes arose through convergent evolution [25]. Despite their common origin, eukaryotic MCs display impressive variation in sequence that is reflected in structure, architecture, interaction, and cage formation differences [25]. Because of this extreme sequence divergence, MC proteins can only be poorly aligned and phylogenetic analyses have been so far intractable, impeding any direct evaluation of the OPH for those proteins. Better models and programs might solve this issue in the future.

Thereby, the PCH proposes that acquisition of the first MC protein endowed this organism with the important evolutionary advantage of being able to manipulate its membrane. Duplication and divergence of this initial MC protein led to diversification and subfunctionalization of the newly formed membrane compartments. This view has been extended, and confirmed by many subsequent studies, and provided a mechanism that unites membrane deformation activities in vesicle coats, the flagellum, and the nuclear envelope [25,26]. However, MC proteins are necessary but not sufficient to the trafficking system and they interact with additional proteins to ensure their function. In keeping with the OPH, the PCH should be extended to the partners of the MC proteins.

Thus, those two complementary hypotheses shed light on related aspects of eukaryotic ES origin and early evolution. The PCH proposes that acquisition of the ancestral MC and associated proteins marked the birth of the eukaryotic ES, while OPH states that the duplication and divergence of those proteins led to the morphological differentiation and functional diversification of the developing eukaryotic ES.

## 3. Our Proposal

Joining both PCH and OPH allows reconstructing the ancestral states of the pre-eukaryotic ES. Based on this, we moved backward in time to propose a possible organisation of the ancestral eukaryotic ES. Those two theories, derived independently, predict that the ancestral eukaryotic ES showed reduced complexity, and was composed of fewer proteins organised into fewer compartments.

### 3.1. Both OPH and PCH Predict an Ancestral Eukaryotic ES with Simpler Organisation, Fewer Proteins and Less Specialized Function

The combination of PCH and OPH suggests that going back in time from a fully differentiated ES, a reduction in the number and divergence of proteins as well as a reduction in the differentiation states of the organelles defined by those proteins would be found, *i.e.*, fewer proteins and fewer functionally differentiated compartments forming a less developed ES. Pushed to the extreme, this reasoning would eventually lead to the prediction of an ancestral minimalistic ES with a single copy of the original MC protein and its partners. However, the questions concerning the organisation and function of this minimalistic ES remain unsolved.

### 3.2. What Shape Could the Ancestral Eukaryotic ES Have Presented?

We propose that the ancestral ES was composed of cytoplasmic membrane invaginations, taking the shape of various tubules and vesicles, loosely connected in a tubulovesicular network (TVN). A TVN is one organizational step beyond the vacuole but is less complex than a fully differentiated ES which requires the development of regulatory, traffic targeting and membrane fission and fusion mechanisms. It is mechanistically easier to expand existing membrane structures, either vacuoles or invaginations, than to separate them. In addition, membrane surface increase (e.g., by lipid overproduction) inside an expansion limited volume (e.g., by peptidoglycan constriction) is likely to result in infolding(s) of the exceeding membrane. Similarly, the development of lipids with different properties, like sterols, might have changed the characteristics of the membrane, leading to increased propensity of invaginations. This conceptually simpler system does not require the development of membrane fission and fusion mechanisms. One prediction of this theory is that it is possible that a prokaryote with membrane fission and fusion system exists and is yet to be discovered. Moreover, a list of various questions remains. How are vesicles and tubules connected? How is the transfer between connected pseudo-compartments regulated?

Thus, the combination of OPH and PCH is compatible with the proposal that the ancestral state of the ES might have been a simple undifferentiated membrane system sustained by a single multi-protein complex that included the MC protein and its partners. In this undifferentiated state, it is plausible that the membranes would adopt a conformation of connected tubules and vesicles organized in a functionally undifferentiated TVN. Importantly, TVNs are observed in various current organisms, providing glimpses of what the ancestral TVN might have looked like.

### 3.3. Tubulovesicular Network in Modern Organisms

We now argue that diverse TVN organisations are observed in current organisms in the three domains of life, showcasing some of the possible conformations of membranes in TVNs. Those include the bacteria *Gemmata obscuriglobus*, the archaea *Ignicoccus hospitalis* and possibly various eukaryotes like *Giardia lamblia*, *Entamoeba histolytica*, and microsporidia.

**Figure 2:**
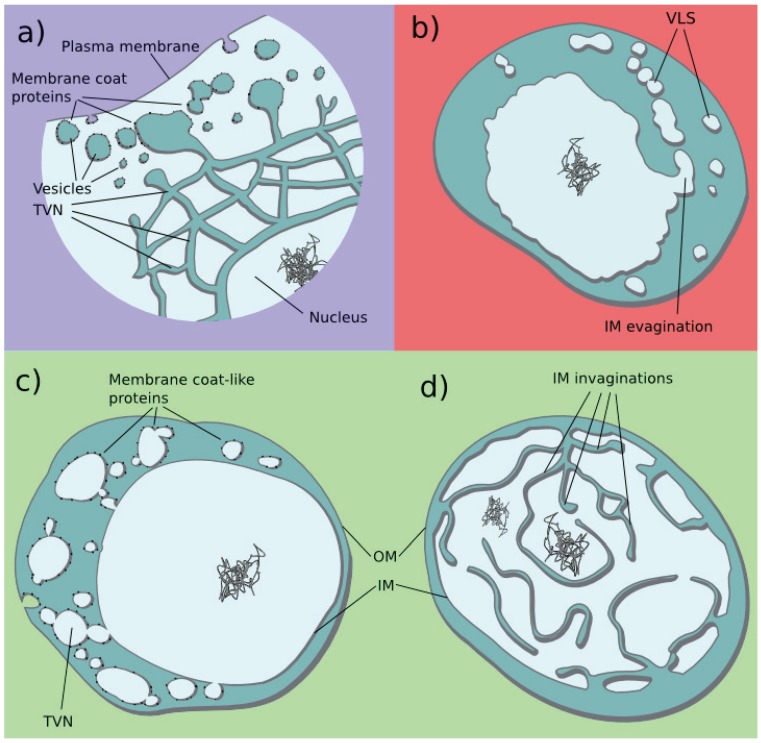
Various types of TVNs in the three domains of life. Schematic of various endomembrane systems are presented. (**A**) *Giardia lamblia* is representative of atypical eukaryotic ES that might be organized as TVNs (only a portion of a cell is represented). Endocytosed proteins are degraded by proteases in the TVN. Membrane fusions between vesicles and the plasma membrane, between vesicles and the TVN are dynamic, redraw based on [42]; (**B**) *Ignicoccus hospitalis* with periplasmic IM evaginations, tubules and vesicles-like structures (VSL) are most likely connected, inspired by [36]; (**C**,**D**) *Gemmata obscuriglobus* cellular membrane organization; (**C**) Cell type with periplasmic TVN, internalized proteins are possibly captured by protein-coated vesicles, from [34]; (**D**) Cell type with cytoplasmic membrane invaginations, based on [33]. Membrane coat-like proteins are represented by dark dots, only in (**A**) and (**C**). DNA is represented as string. Background is blue for eukaryote, red for archaea and green for bacteria.

*G. obscuriglobus* is a bacteria belonging to the Planctomycetes phylum [27]. Members of the Planctomycetes and related bacteria of the Verrucomicrobia phylum stand out amongst bacteria for the development of their cellular membranes [28,29]. Various analyses have focused on those peculiar intracellular membrane systems [30,31,32]. In particular, two cell types have been described in *G. obscuriglobus* based on the organisation of their cellular membranes. In one cell type, inner membrane (IM) invaginations fill the cytoplasm (Figure 2) [33]. In the other cell type, a network composed of connected membrane vesicles and tubules is present in the periplasm of the cell (the space located between the IM and the outer membrane (OM) of bacteria) [34]. This network appears to link the outside of the cell to the cytoplasm through a network of connected vesicles and tubules. The contents of neighbouring vesicles sometimes appear different in terms of colour and density, suggesting regulation of exchange between compartments [34]. Thus, a complex membrane system that is organized as a TVN in the periplasm of these cells is present in at least one bacterial species.

*I. hospitalis* is an archaeal lineage deeply branching within the family of the Desulfurococcaceae [35]. Unlike most archaea, members of the Ignicoccus genus are surrounded by two membranes instead of one (Figure 2). Similarly to one of the *G. obscuriglobus* cell types, tubular extensions of the IM that extend in the periplasm of *I. hospitalis* have been described [36]. Vesicles have also been observed in the periplasm of this archaea [37]. Intraperiplasmic tubules and vesicles are likely to be connected in the periplasm. Therefore, a particular TVN-like membrane organisation is present in archaea too. Similar organization appears to be present in another unrelated archaea phylum, suggesting that this phenomenon might be more prevalent than expected [38].

Thus, membranes organisation in a TVN are present in both prokaryotic domains. Our knowledge of those unusual system is however limited, and it is important to describe them in more details and to expand our exploration of biodiversity.

Some eukaryotes display an atypical ES of reduced morphology. The exact organisation of their membrane systems is still under investigation, but it has been suggested that their atypical membrane organisations might be interpreted as diverse TVN morphologies. They are described using various terms; Golgi-derived branching or varicose tubules, as a tubular network (although no vesicles are formed) in microsporidia [39]; ER-derived continuous reticular network in *E. histolytica* [40,41]; and a contiguous ER and endosome/lysosome compartment continuous with the nuclear envelope in *G. lamblia* (Figure 2) [42]. The *G. lamblia* ES has been interpreted as “an ER-like tubulovesicular compartment, which itself can dynamically communicate with clathrin-containing vacuoles at the periphery of the cell to receive endocytosed proteins” [42]. In addition, the TVN of *G. lamblia* serves as a site of protein synthesis but also of degradation of material from the outside [42] supporting our proposal of multi-functionality of the ancestral TVN.

Despite being still discussed, the reported morphologies of the ES of *G. lamblia*, *E. histolytica*, and microsporidia illustrate the possibility of a TVN in extant eukaryotes. Similarly to the prokaryotic ones, more analyses at higher resolutions are required to determine the precise organisation of those membrane systems.

There is no evolutionary relationship between those organisms, nor do we imply one with the ancestral eukaryotic ES. Those particular eukaryotic cell plans are most likely derived by reductive evolution from a complex ancestor with a ‘classical’ eukaryotic ES [19,41,43]. They are unlikely to be deep branching or ancestral. On the other hand, the archaeal and bacterial TVNs are located in the periplasm, while the eukaryotic ones are in the cytoplasm. They are thus topologically inverted, which is a crucial difference. We only highlight them as illustration of current TVNs.

However, the *G. obscuriglobus* membrane system displays additional intriguing features.

Firstly, genes coding for proteins with the MC architecture had been detected in the genomes of various members of the Planctomycetes-Verrucomicrobia-Chlamydiae (PVC) superphylum, as well as Bacteroidetes [44]. No such genes have been detected in archaeal genomes so far, including that of *I. hospitalis* [45]. In addition, in *G. obscuriglobus*, at least one MC-like protein is located in the periplasm in tight interaction with the membranes of the TVN [34]. This represents a so far unique molecular link between a bacterial and eukaryotic ES. Nonetheless, those MC-like proteins have only been identified using a structural approach and no sequence similarity can be detected between the eukaryotic and bacterial MCs [44,46]. This does not mean that the bacterial and eukaryotic MC proteins are not related, as the same argument, the lack of sequence similarity, applies between eukaryotic MCs, whose homology is now accepted [25]. The lack of sequence similarity only means that the evolutionary relationship, or lack thereof, between eukaryotic and bacterial MC proteins cannot be determined based on this criteria only [46,47]. A possible argument against the evolutionary relationship between the two set of proteins is their inverted localisation, where the bacterial ones are located in the periplasm of the bacteria, and the eukaryotic ones in the cytoplasm. Disregarding the possible, or not, evolutionary relationship between eukaryotic and bacterial MC proteins, the implication of MC-like proteins in a bacterial TVN is further evidence in favour of the PCH, supporting the role of this particular protein architecture in membrane manipulation. If this MC architecture has appeared more than once during evolution is a fascinating question that is still unanswered.

Secondly, eukaryotic ES origin is linked to the apparition of the endocytic phenomenon, so far restricted to eukaryotes. Intriguingly, protein internalisation and degradation has been reported to occur in *G. obscuriglobus* [48]. Based on the energy-dependency and competition-sensitivity of this phenomenon, it has been suggested that it is similar to the eukaryotic process of endocytosis [48]. However, the presence of a TVN that links the OM to the IM in this organism [34] might provide an alternative explanation, namely a facilitated diffusion of proteins through the membranous network that links the exterior of the cell to the cytoplasm via the periplasm. Thus, the bacterial TVN might be linked to a phenomenon that was previously only found in eukaryotes and had been key to their development. Whatever the mechanism, whole protein internalisation and internal degradation is present in *G. obscuriglobus* too.

In summary, different flavours of TVN are present in the three domains of life, bacteria, archaea, and eukaryotes, showcasing, each in their different ways, what ancestral TVN(s) might have looked like. One of those organisms also displays features of MC proteins in contact with the membranes and whole protein internalisation and degradation.

### 3.4. What Function Could the Ancestral Eukaryotic ES Have Contained?

A reduction in the number and differentiation of the compartments as well as in the number of paralogue proteins involved in maintaining them might be linked with less functionally specialized organelles. Thus, the ancestral TVN might have been multi-functional, with the precursors of each function differentiated in the subsequent steps present in embryonary forms in less specific proteins and compartments. The multiple functions of compartments not detected in atypical eukaryotic ES are actually found in the same compartment [42].

#### Function of Current TVNs

We can use current TVNs to speculate about the function of such an ancestral membrane organisation. However, caution is required, as current TVNs are derived and the current function might not be related to the ancestral one. We will not use the eukaryotic ones as they are clearly the result of reductive evolution from a more complex ancestor and thus have an evolutionary history that might not be compatible with this type of inference. The TVN of *I. hospitalis* is still poorly characterised and little data is available about its function. However, localisation of one of the subunit of the classical ATPase in the OM suggested that this membrane organization unique in archaea is related to energy acquisition [49]. This is however not the TVN, but the OM of the archaea.

Slightly more information is available for the bacterial TVN. In *G. obscuriglobus,* the TVN has been linked to the phenomenon of protein internalization and degradation reported previously in this organism. This phenomenon suggests that the initial function of the ancestral eukaryotic TVN might have been related to internal feeding greatly improving aerobic digestion by limiting external waste. This agrees with phylogenetic reconstruction of the Rab paralogs into two clades corresponding to endocytic (‘in’) and exocytic (‘out’) functions and predating the emergence of specialized organelles [6]. Thereby, despite the lack of information, very crude inference suggests that the function of the ancestral TVN might have been related to internalization and externalization.

Based on energetic considerations, it has been suggested that eukaryogenesis was only possible subsequently to the acquisition of the mitochondria, implying a ‘mitochondria-early’ scenario [50]. In particular, the development and maintenance of a developed ES is speculated to have been energetically costly. The observation of a TVN in two prokaryotes devoid of energy producing organelles (a symbiont has been described in *I. hospitalis*, but it is not required for growth [37]), suggests that the acquisition of the energy powering organelle of the cell might have been required at later stages of evolution, which is more compatible with a ‘mitochondria-late’ scenario. Thus, whatever the energetic justification, those prokaryotic ESs demonstrate that steps towards development of a complex ES are possible without the acquisition of the mitochondria. This means early steps of eukaryogenesis, *i.e.*, the beginning of development of some eukaryotic features, are possible, up to a certain point, without the energetic contribution of the mitochondria. Such an example is the development of the ES in a TVN organisation. Further development is likely to require the mitochondria to fulfil higher energetic needs [50].

### 3.5. A Coherent Model of Complex Intracellular Membrane System Evolution

**Figure 3:**
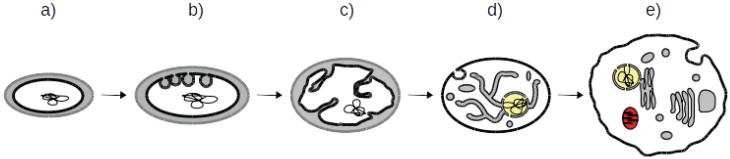
Model for endomembrane system development with TVN as intermediary step. The figure represents schematics of putative intracellular membrane organisations, showcasing the different steps of the proposed hypothesis. Schemas not to scale. DNA is represented as a (wrapped) string. Prokaryotic periplasm is coloured in light grey and cytoplasm in white. Cytoplasmic and inner membranes (IM) are in black, outer membranes (OM) are in light grey. Mitochondrial and nuclear spaces are red and yellow, respectively. (**a**) diderm prokaryotes (Step one); (**b**) prokaryotes with IM vesicles (Step two); (**c**) prokaryotic intracellular membrane organisations, with IM invaginations (Step three); (**d**) intermediary organism with a multifunctional TVN (Step four); (**e**) ‘classical’ eukaryote with a fully developed and functionally differentiated endomembrane system (Step five).

Both molecular phylogenetic analyses [51,52,53] and evidence from the fossil record [54,55,56] support the notion that prokaryotes predated eukaryotes. This evidence suggests that the eukaryotic cell must have arisen from a state resembling that of a prokaryote; that is, that the acquisition of organelles and complex cellular machineries in eukaryotes must be explained from a cellular state lacking most membrane organisation. Consequently, most credible scenarios of eukaryotic ES origin start from a prokaryotic stage [53,57,58]. However, important gaps in membrane organisation are still present and have been used to criticize those scenarios [59]. The prediction of a multi-functional TVN as the ancestral eukaryotic ES refines current autogenous theories of eukaryotic ES development and fills the gap between prokaryotes and eukaryotes by providing plausible intermediate organisations (Figure 3).

Given the arguments presented above, a coherent model of ES evolution based on autogenous development from a ‘simple’ prokaryotic one can be proposed. This model proposes a gradual development of the intracellular membranes, where each step is the result of simple increments over the previous one.

This broad-brush scenario starts with a 'simple' membrane organisation, as observed in current prokaryotes, showing little to none particular features of their membranes (Step one; Figure 3). As our model deals with the cytoplasmic or inner membrane (IM), the number of additional membranes, one or none as observed in current diderms or monoderms [60], has little relevance, although a monoderm would not require an additional step of loss of the OM. Limited invaginations of the IM lead to vesicles or saccules in the cytoplasm still attached to the IM and thus filled with periplasmic content (Step two). Next, extensions of these invaginations either towards the cytoplasm or towards the periplasm form a more developed organisation of membranes inside the cell volume (Step three). The developed IM invaginations represent true internalisation of the periplasm inside the cytoplasm since they are still connected to the IM and thus, their lumens are continuous with the periplasm, mainly filled with periplasmic material, although local synthesis through the membrane could lead to increase local concentration of different compounds. This step was probably associated with the apparition of the initial MC protein and partners. Duplication and divergence of those proteins would be associated with the separation of those compartments as well as with their sub-functionalisation. At this point the invaginations would become a TVN (step four); how this is related to the duplication of the proteins sustaining it is unclear and probably overlap in a blurry manner. Complete separation of the compartments and their functional specialisation as well as separation of the ES from the cytoplasmic membrane would follow (step five). Notice however, that even in current eukaryotes, connections are observed between some of the compartments [9]. The acquisition of the mitochondria likely happened close to the end of this transformational process and marked the birth of the eukaryotic realm (although earlier origin is also possible [1,50]).

This scenario is in agreement with and refines related scenarios of nucleus evolution, where the ‘sophisticated prokaryote’ and the ‘proto-eukaryotes’ go through stages of membrane development, before the development of the nucleoporins that are compatible with a multi-functional TVN [26]. This is also compatible with remote homology detection of nucleoporins in most eukaryotes as it implies that a fully functional ES, including most organelles and a complete NPC, was already present in the LECA [61], since the TVN as presented here is a pre-eukaryotic feature before the specialization of the organelles, including the nucleus. It is possible that one or more of the invaginations of the organizing ES, mediated by one of the copies of the MC proteins, wrapped around the genetic material of the proto-eukaryote. Evolution into a modern nuclear envelope fenestrated by nuclear pores is conceptually a small step.

Was there an archaeal or bacterial ancestor to the ES? It is increasingly accepted that eukaryotes and archaea share a common ancestor [51]. There is however disagreement about whether eukaryotes and archaea are sister phyla or if eukaryotes are derived from an archaeal ancestor [62]. The observation of TVN in archaea suggests that evolution of the ES might have started in this domain. This possibility is additionally supported by the existence of precursors of eukaryotic features in prokaryotes [63]. There is currently not enough information to speculate on whether eukaryotic origin can be pushed back to bacteria.

This scenario is not a revival of the archezoa hypothesis which stated that amitochondriate eukaryotes diverged before the origin of mitochondria [64]. It is now clear that all such eukaryotes are derived from an ancestor with mitochondria and the archezoa hypothesis is now considered by most scholars to be flawed [65]. The LECA, ancestor of all eukaryotes, possessed a mitochondria and an ES composed of functionally differentiated and spatially separated compartments. Our scenario deals with steps before the LECA and proposes that the ancestral ES might have been organized as a TVN, before spatial and functional divergence and specialization of the organelles.

We argue that each step of this scenario is supported by the existence of cellular membrane organisations observed in various organisms. In addition to the TVN developed above, those steps are discussed below (Table 1).

### 3.6. Current Organisms Illustrate Our Scenario

Most current bacteria do not show any particular membrane organisation (Step 1). Some bacteria have been reported to release signalling molecules that are packaged in vesicles budding off from the OM, but these appear to be exceptional cases [66]. Other bacteria can be stimulated by particular conditions or mutations to invaginate their cytoplasmic membrane into extensive intracytoplasmic membrane inclusions and vesicles [67,68]. Although these invaginations do not appear to be present under natural conditions, they illustrate that those organisms have the capacity to elaborate membrane structures.

**Table 1:** Steps of endomembrane development. The table lists the steps of the proposed ES development scenario with the name, features and examples that are illustrated. The figures represent schematics of intracellular membrane organisations in the three domains of life. DNA is represented as a string. Prokaryotic periplasm is coloured in light grey, cytoplasm in white and anammox in dark grey. Cytoplasmic and inner membranes (IM) are in black, outer membranes (OM) are in light grey. Mitochondrial and nuclear spaces are red and yellow, respectively. (Step 1) mono- (left) or diderm (right) prokaryotes, including bacteria and archaea; (Step 2) prokaryotes with IM-derived vesicles, magnetotactic (left), photosynthetic (middle) and anammox (right) bacteria; (Step 3) prokaryotic intracellular membrane organisations, with IM invaginations, such as *G. obscuriglobus* cell type 1 (left) and *Planctomycetes limnophilus* (right), most vesicle-like structures are likely IM evaginations cut perpendicularly to the plan of the membrane; (Step 4) intermediary organisms with a multifunctional TVN, such as *G. obscuriglobus* (cell type 2) (left) and *G. lamblia* (right), most tubules and vesicle-like structures are likely connected, forming a TVN; (Step 5) ‘classical’ eukaryote with a fully developed functionally differentiated endomembrane system. Schemas not to scale. (A) archaea, (B) bacteria, (E) Eukaryotes.

Steps	Name	Features	Examples	Illustrations
1	Simple	None (monoderm or diderm)	Prokaryotes (most of them, both archaea and bacteria)	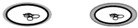
2	Vesicles	Vesicle or Saccules	Magnetotactic; photosynthetic and anammox bacteria	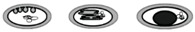
3	Invaginations	IM invaginations	PVC bacteria	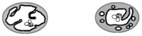
4	TVN	Connected tubules and vesicles	*G. obscuriglobus* (B), *Ignicoccus hospitalis* (A), and *Giardia lamblia* (E)	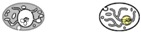
5	Developed	Functionally specialized and spatially separated	Eukaryotes (most of them)	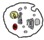

More developed internal membrane morphologies have been observed under native conditions in some bacteria (Step 2) [69]. Most are simple invaginations of the cytoplasmic membrane that define a few pseudo-compartments that are still connected to it. Magnetotactic bacteria enclose magnetic crystals in a few vesicles connected to the plasma membrane. Photosynthetic bacteria sometimes present an impressive network of sacculi, in which they localise the photosynthetic system, forming the thylakoids.

Bacteria from the PVC superphylum arguably present the most diverse prokaryotic intracellular membrane organisations, with extensive morphologies based on one or a few invaginations of the cytoplasmic membrane towards the inside of the cell, or evaginations of the outer-membrane towards the outside (Step 3) [29]. Moreover, the planctomycetes phylum possibly contains species with one of the few true prokaryotic organelles, if organelles are defined as structural compartments that are enclosed within their own lipid bilayers separated from other cellular membranes to isolate a specific function. The anammox (anaerobic ammonium oxidation) planctomycetes are a monophyletic deep branching group. They show an unusual intracellular membrane organisation, which contains an energy-producing compartment, the anammoxosome, that is located in the cytoplasm and appears to be separated from the cytoplasmic membrane [70]. As discussed above, various TVNs are observed in current organisms in the three domains of life (Step 4). Exploration of this phenomenon and deeper analysis of the described ones are important steps.

Current eukaryotes represent the so far ultimate development and functional differentiation of ES (Step 5). The existence of a developed ES is strongly linked to the appearance of the eukaryotic endosymbiotic organelles, the mitochondria and the chloroplasts [54]. Although not sufficiently explored in prokaryotes, endosymbiosis has also been described in Gamma- and Alpha-proteobacteria, as well as archaea [61]. Interestingly, *I. hospitalis* can harbour a symbiont, in this case, another archaea, the Nanoarchaeota, which can only grow outside of *I. hospitalis* and remains attached to its OM [34]. These illustrate another important evolutionary event linked to internal membranes in prokaryotes, the enslaving of another engulfed prokaryote. The occurrence of this phenomenon in prokaryotes should also be explored further.

## 4. Discussion

In order to falsify this model, it would be important to characterize in more detail and with increased resolution the putative TVNs observed in current organisms, as well as to discover new ones. In this sense, increasing our evolutionary cell biology and biodiversity exploration efforts is important. A higher resolution in the phylogeny of the eukaryotic proteins related to endomembrane resolution might improve our reconstructions of those crucial events. The results of such investigations are bound to reveal fascinating details with implications for molecular and cellular biology as well as phylogeny and evolution, eventually leading to insights into early stages of evolution of complex intracellular membrane systems such as that found in our own cells.

## 5. Conclusions

The combination of OPH and PCH suggests that the ancestral eukaryotic ES might have been composed of a multi-functional and undifferentiated TVN. Although this is only one of the possibilities, this conclusion is supported by, among other things, the evolutionary advantage of external substances internalisation capability through the TVN. In addition, various membrane organisations in a TVN are observed in current organisms in the three domains of life. Based on this, we have proposed a coherent model of complex endomembrane system development from an undeveloped one that falls in the ‘mitochondria-late’ series of scenarios.

## Abbreviations

ES: Endomembrane system
OPH: organelles paralogy hypothesis
PCH: protocoatomer hypothesis
MC: membrane coat
TVN: tubulovesicular network
